# *Hot topic:* Influenza A H5N1 virus exhibits a broad host range, including dairy cows

**DOI:** 10.3168/jdsc.2024-0638

**Published:** 2024-09-30

**Authors:** Salman L. Butt, Mohammed Nooruzzaman, Lina M. Covaleda, Diego G. Diel

**Affiliations:** Department of Population Medicine and Diagnostic Sciences, College of Veterinary Medicine, Cornell University, Ithaca, NY 14853

## Abstract

The widespread circulation of highly pathogenic avian influenza (HPAI) H5N1 clade 2.3.4.4b in wild birds in North America since late 2021 has resulted in multiple outbreaks in commercial and backyard poultry leading to major economic losses. Since the emergence of the virus in North America, multiple reassortment events have led to the emergence of many new variant genotypes that have been isolated from wild birds, with several viruses spilling over into poultry and other terrestrial and aquatic mammalian hosts. Notably, the most recent emerging HPAI H5N1 reassortant genotype B3.13 spilled over into dairy cattle (*Bos taurus*), resulting in unprecedented efficient transmission of the virus in this species, the first case of sustained transmission in a mammalian species. The transmission pathways involved in the spread of the virus from its first detection in Texas to several other states are complex. However, movement of subclinically infected cattle likely played a major role in virus spread. Infection in dairy cattle is characterized by the virus's tropism for milk-secreting cells in the mammary gland, leading to high viral load and shedding in milk. Replication of the virus in milk-secreting cells results in destruction of infected cells leading to severe viral mastitis, which is characterized by marked changes in milk quality (altered consistency and color) and pronounced decline in milk production by clinically affected animals. Here, we provide an overview of the HPAI H5N1 panzootic virus and discuss its host range and the current knowledge of its pathogenesis in the new bovine host.

## Highlights

• Dairy cattle are highly susceptible to HPAI H5N1 virus infection and replication.
• Movement of subclinically infected cattle likely plays a major role in virus spread.
• The HPAI virus infection leads to severe viral mastitis and poor milk quality.
• The virus's tropism for the mammary gland leads to high viral load and shedding in milk.
• Raw milk has an important role in interspecies virus transmission from affected dairy cattle.

## Graphical Abstract

**Figure:**
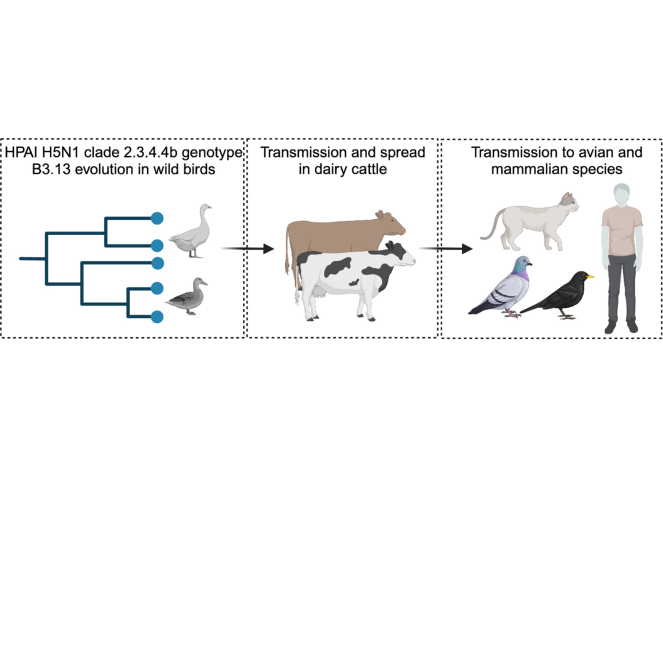
**Summary:** The widespread circulation of highly pathogenic avian influenza (HPAI) H5N1 clade 2.3.4.4b in wild birds in North America has resulted in multiple outbreaks in commercial and backyard poultry. The emergent HPAI H5N1 reassortant genotype B3.13 spilled over into dairy cattle, resulting in unprecedented transmission of the virus in this species: the first case of sustained transmission in a mammalian species. The transmission pathways involved in the spread of the virus are complex; however, the movement of subclinically infected cattle likely played a major role in virus spread. Replication of the virus in milk-secreting cells results in destruction of infected cells, causing severe viral mastitis and, consequently, poor milk quality and a pronounced decline in milk production by clinically affected animals. Here, we provide an overview of the HPAI H5N1 panzootic virus and discuss its host range and the current knowledge of its pathogenesis in the new bovine host. Graphical abstract created with BioRender.com.

Influenza A viruses (**IAV**) are important pathogens causing respiratory or systemic infections in multiple host species. These viruses have a single-stranded negative-sense segmented RNA genome and are classified within the family *Orthomyxoviridae*. The IAV genome consists of 8 (08) segments including the hemagglutinin (**HA**), neuraminidase (**NA**), matrix (**M**), nucleoprotein (**NP**), polymerase basic 1 (**PB1**), polymerase basic 2 (**PB2**), polymerase acidic (**PA**), and nonstructural 1 (**NS1**). The classification of IAV into genetic and antigenic subtypes is based on the combination of the hemagglutinin (**HA**) and neuraminidase (**NA**) surface glycoproteins. To date, a total of 19 HA-types (**H1–H19**) and 11 NA-types (**N1–N11**) have been identified and the combination of these gene segments through reassortment results in different IAV subtypes (e.g., H5N1, H7N3, H9N2, and so on) (Webster et al., 1992; Alexander, 2000; Shu and McCauley, 2017). Additionally, avian IAV can be classified into 2 biotypes: highly (**HPAIV**) and low pathogenic avian influenza viruses (**LPAIV**) based on their pathogenicity in chickens (*Gallus gallus domesticus*). At the molecular level, the distinction between LPAI and HPAI lies on the cleavage site of the HA protein, where LPAIV have a monobasic cleavage (PEKQTR/GLF) site containing a single arginine that is recognized by trypsin-like proteases expressed predominantly in the respiratory and intestinal tract. In contrast, HPAIV present multibasic cleavage site (PQRESRRKK/GLF) that is cleaved by ubiquitous furin and furin-like proteases (Stieneke-Gröber et al., 1992; Bertram et al., 2010). The cleavage of HA and tissue distribution and expression of the proteases in host tissues are the major determinants of influenza virus replication and pathogenesis in chickens.

Naturally occurring HPAI viruses belong to either H5 or H7 subtypes and are responsible for high mortality in poultry and outbreaks in mammals including zoonotic spillovers into humans (Abdelwhab and Mettenleiter, 2023). Based on phylogenetic clustering, common ancestors, and their descendants, IAV genome sequences are classified into clades (1.1, 2.2, 2.3, and so on), subclades (2.3.2.1c, 2.3.3.4b, and so on), lineages (Eurasian, American), and genotypes (A1, B1, B3.13, and so on) (Elsmo et al., 2023; Youk et al., 2023).

In the last decade, the HPAI H5N1 clade 2.3.4.4b emerged and spread worldwide, becoming the predominant and most genetically diverse group of IAV. This genetic diversity occurred because of constant genetic and antigenic shift and drift. The error-prone RNA polymerase rapidly generates mutations in the progeny, resulting in a diverse population of viruses. The reassortment of gene segments is another efficient mechanism of genetic evolution in IAV (Ma et al., 2016; Youk et al., 2023), potentially leading to the emergence of entirely new viruses. Although pigs (*Sus scrofa domesticus*) have been considered a mixing vessel, reassortment is not limited to swine, as waterfowl, in particular wild migratory and domestic ducks (*Anas platyrhynchos domesticus*), serve as intermediate mixing vessels where influenza viruses of different subtypes mingle and reassort, generating new viruses that have different antigenic properties and sometimes distinct virulence characteristics and host preferences (Gilbert et al., 2010; Youk et al., 2023). Importantly, migratory wild birds act as carriers capable of long-distance, cross-continental spread of IAV, posing a risk for the introduction of novel HPAI viruses to new geographic regions. Indeed, the 3 recent HPAI outbreaks in the United States occurred after introduction of H5N2 and H5N1 viruses through migratory wild birds (Alarcon et al., 2018; Puryear et al., 2023; Hu et al., 2024 [unpublished data]).

Notably, H5N1 clade 2.3.4.4 became dominant globally from 2014 onward. In 2016, the H5N1 clade 2.3.4.4b caused the largest European epidemic and since then has caused several outbreaks in poultry and wild bird species globally (Alarcon et al., 2018). The Eurasian lineage of H5N2 clade 2.3.4.4 was responsible for the 2014­–2015 outbreaks of HPAI in the United States, which led to the death of more than 50 million chickens, turkeys (*Meleagris gallopavo domesticus*), and other birds. Since 2020, H5N1 clade 2.3.4.4b is expanding its geographic- and host range, leading to an unprecedented number of deaths in poultry and wild birds and posing an ongoing risk of spillover infections into mammals, including humans (World Health Organization, 2023). In 2022 a large outbreak of H5N1 in farmed minks (*Neovison vison*) caused high mortality rates in these animals in Spain, consisting of the first documented case of efficient mammal-to-mammal transmission of the virus (Agüero et al., 2023).

The first transatlantic transmission of H5N1 clade 2.3.4.4b was detected in December 2021 in a free-living gull (*Larus canus*) in St. John's, Newfoundland, and Labrador, Canada. Subsequently, in February 2022, the virus was detected in wild ducks (*Anas platyrhynchos*) in South Carolina, spreading to multiple US states and causing the ongoing 2022–2024 HPAI outbreak that resulted in the deaths or culling of more than 100 million poultry in 48 states (Bevins et al., 2022; Caliendo et al., 2022; USDA-APHIS, 2024). A unique feature of the current 2022–2024 HPAI outbreak is the frequent spillover of the virus to several wild terrestrial and aquatic mammalian species, with at least 22 species infected to date (USDA-APHIS, 2024). Among affected mammals, harbor seals (*Phoca vitulina*) (Liang et al., 2023), red fox (*Vulpes vulpes*) (Cronk et al., 2023; Bordes et al., 2023), and striped skunks (*Mephitis mephitis*) are the prominent species that were infected during 2022–2023 (Elsmo et al., 2023). Harbor and grey seals (*Halichoerus grypus*) affected in New England in the United States showed high mortality with approximately 330 animals dying from the infection (Puryear et al., 2023). Importantly, in early March 2024, a goat (*Capra aegagrus hircus*) kid from Minnesota tested positive for HPAI H5N1 genotype B3.9 (Schnirring, 2024), representing the first detection of the virus in a ruminant species. Notably, soon thereafter spillover of an H5N1 virus belonging to a reassortant genotype B3.13 was detected in dairy cattle (*Bos taurus*) in Texas with further spread to other states and evidence for cow-to-cow transmission documented for the first time in mammals (Burrough et al., 2024; Caserta et al., 2024) (Figure 1).

**Figure 1:**
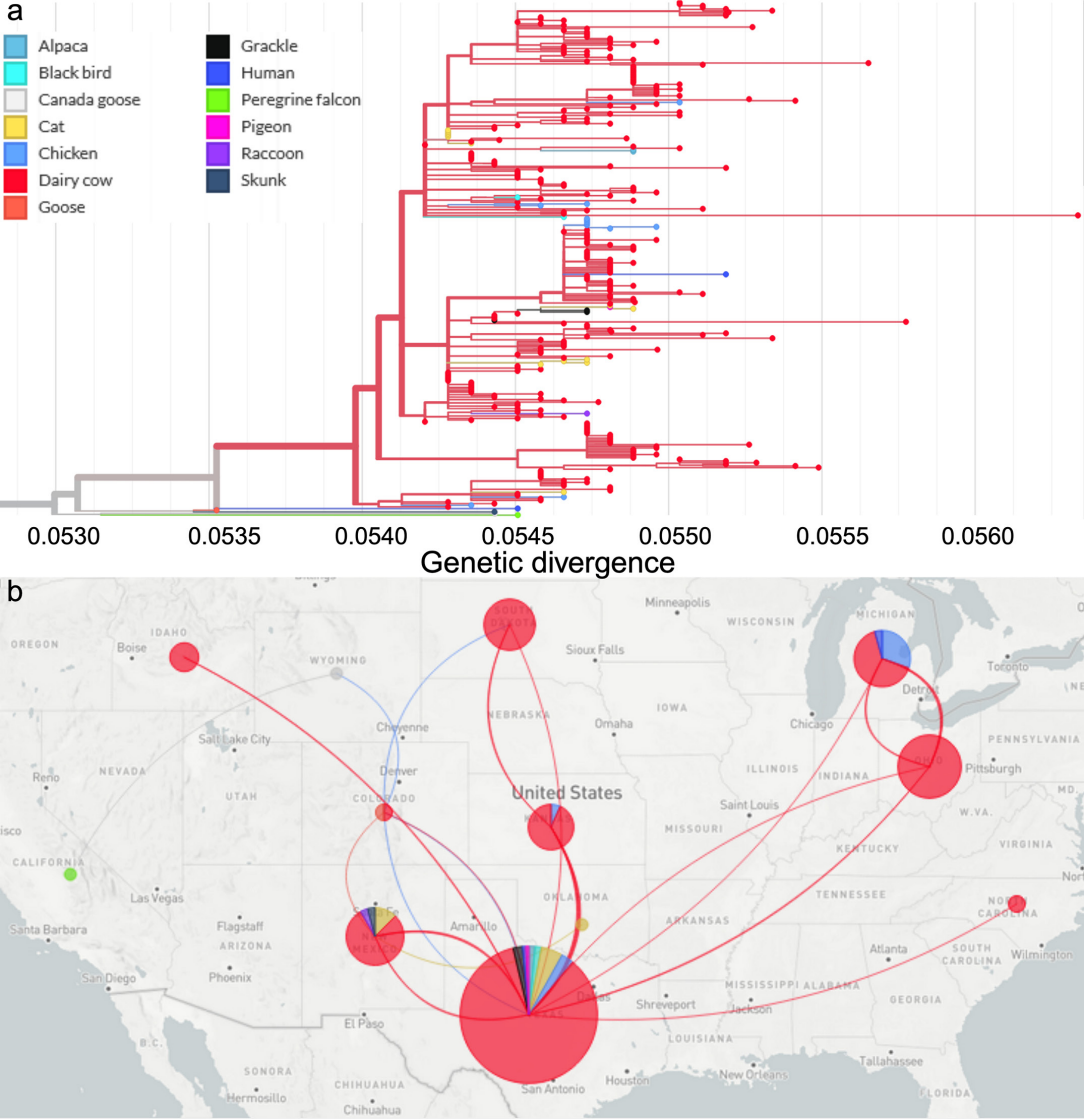
Phylogenetic analysis of HPAI H5N1 clade 2.3.4.4b genotype B3.13 circulating in the United States from 2023 to 2024 in avian and mammalian animal species. (a) Genetic divergence of HPAI H5N1 genotype B3.13 circulating in wild, domesticated avian, and mammalian species. Different node colors represent host species. (b) Interstate spread of genotype B3.13 after first detection in Texas. Lines represent anticlockwise directionality of virus dispersal.

Since the first detection of HPAI H5N1 in the North America in early 2022, surveillance of migratory wild birds revealed widespread circulation of HPAI H5N1 among avian species. Notably, multiple genotypes—reassortants of Eurasian and American lineages of H5N1—rapidly emerged and circulated in wild birds across states. The fall wild bird migration season (August to November) of 2023 likely contributed to numerous reassortment events of H5N1 in wild birds, ultimately resulting in the emergence of the H5N1 B3.13 genotype. The earliest report of H5N1 B3.13 was in November 2023, found in a Canada goose (*Branta canadensis*) from Colorado, followed by detections in a Canada goose from Wyoming and a peregrine falcon (*Falco peregrinus*) from California in January of 2024. The spring wild bird migration (January to April) may have led to widespread circulation of H5N1 B3.13 in wild birds, facilitating its spillover to other species, and eventually dairy cattle (Burrough et al., 2024; Caserta et al., 2024).

The transmission pathways of H5N1 to dairy cattle and its subsequent spread between cows are complex and not yet fully understood. While wild birds are likely the primary source of the virus, gaps in surveillance complicate definitive conclusions regarding the host species responsible for transmitting the virus to cattle. The mode of transmission also remains unknown. Given the respiratory nature of IAV, a plausible entry pathway would be through the respiratory route originating from direct contact with an infected host or indirect contact with the virus via contaminated environment, feed, or water. Another plausible route, considering the virus's tropism for the mammary gland (Burrough et al., 2024; Caserta et al., 2024) and its ability to establish infection after intramammary inoculation (Baker et al., 2024 [unpublished data]; Halwe et al., 2024 [unpublished data]), would be direct infection of the mammary gland through the teat canal. This could occur, for example, following contamination of the skin of the udder or teats (or both) with virus present in the environment (floors, bedding, and so on), leading to entry into the mammary gland during milking.

Current evidence linking interstate movement of cows from HPAI-affected farms to new premises, including dairies located thousands of miles away, suggest efficient cow-to-cow transmission, leading to disease outbreaks (Nguyen et al., 2024 [unpublished data]; Caserta et al., 2024). In this context, subclinically infected animals may play a role on virus dissemination (Caserta et al., 2024). Abundant infectious H5N1 virus shedding in milk from affected cows (Caserta et al., 2024) may contribute to multiple routes of virus transmission between cows and from cows to other susceptible species (Caserta et al., 2024), including humans (CDC, 2024), in affected farms. The most direct evidence linking raw milk to H5N1 transmission comes from cats (*Felis catus*) that were found dead in multiple dairy farms during H5N1 outbreaks in dairy cattle (Burrough et al., 2024; Caserta et al., 2024). Comparative genomic sequences of the viruses detected in dairy cattle and cats in these farms revealed a close genetic relationship between the viruses detected in these species, which was supported by epidemiological information indicating the practice of feeding raw milk to cats in these farms (Burrough et al., 2024; Caserta et al., 2024). These observations underscore the need for proper disinfection or treatment of raw milk before disposal or feeding to other animals. Additionally, aerosolization of milk in the milking parlor during milking or cleaning procedures may play a role in H5N1 transmission to cows, cats, birds, and humans. High levels of environmental contamination resulting from aerosolization in the milking parlor or disposal of raw waste milk from affected animals could lead to contamination of fomites (vehicles, trucks, equipment, shoes, and clothing of farm personnel, and so on), which could then vector the spread of the virus within affected farms and from affected farms to other farms (including poultry farms) in the region. These complex transmission pathways underscore the need for strict biosecurity practices to minimize spread and transmission of H5N1 virus from affected dairy farms (Figure 2).

**Figure 2:**
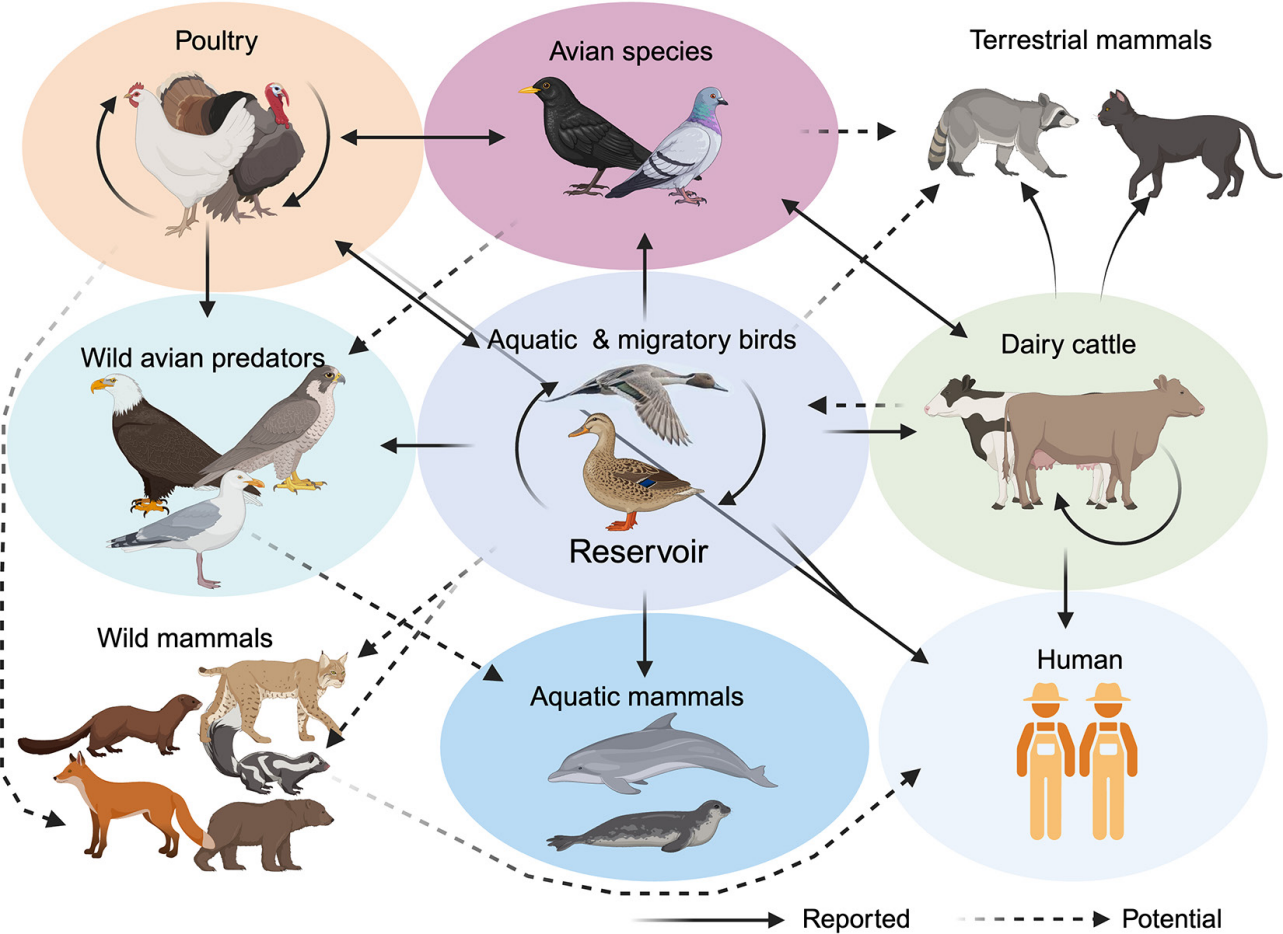
Highly pathogenic avian influenza H5N1 clade 2.3.4.4b virus infection in avian, mammalian, wild, and domestic hosts. Avian influenza transmission flow from the natural reservoir (aquatic and migratory birds) to poultry, humans, and other animal species.

Species susceptibility and spillover of IAV is linked to the expression and distribution of the virus entry receptor, sialic acid (**SA**). Expression, anatomic distribution, and accessibility of SA receptors are key in determining the predilection site of virus replication. The avian origin IAV present preferential tropism to N-acetylneuraminic acid linked to galactose in an α2,3 (avian) configuration. However, human origin IAV preferentially recognize N-acetylneuraminic acid linked to galactose in an α2,6 (human) configuration (Suzuki et al., 2000). Interestingly, the avian SA-α2,3 receptor has 2 additional species-specific forms: SA-α2,3-gal-β1,4 expressed in chicken, and SA-α2,3-gal-β1,3 expressed in ducks (Kuchipudi et al., 2009; Wang et al., 2013). In humans (*Homo sapiens*), higher levels of SA-α2,6 receptor expression are observed in the upper respiratory system and ileal epithelial cells, whereas expression of SA-α2,3 receptors is restricted to the lower respiratory and digestive systems (Trebbien et al., 2011; França et al., 2013; Wu et al., 2014). Importantly, in pigs, SA-α2,6 (human) receptor is expressed throughout the respiratory system, whereas SA-α2,3 (avian) receptors are present in the lower respiratory tract (bronchioles and alveoli) (Trebbien et al., 2011). This makes pigs susceptible to infection with both human and avian influenza viruses, thus potentially serving as mixing vessels where different IAV subtypes can co-infect, leading to new reassortant viruses.

Recent studies in bovines revealed that this species expresses SA-α2,3 (avian) receptors in the trachea, whereas SA-α2,6 (human) is not expressed in this organ (Kristensen et al., 2024 [unpublished data]). Notably, SA-α2,6 (human) and SA-α2,3 (avian) including SA-α2,3-gal-β1,4 (chicken) and SA-α2,3-gal-β1,3 (duck) subtypes are expressed in the mammary gland of dairy cattle (Kristensen et al., 2024 [unpublished data]; Nelli et al., 2024), which could have led to the increased tissue tropism and high levels of virus replication in the mammary gland (Baker et al., 2024 [unpublished data]; Burrough et al., 2024; Caserta et al., 2024; Nelli et al., 2024). This led to the hypothesis that dairy cattle may also serve as a mixing vessel for IAV (Kristensen et al., 2024 [unpublished data]).

The pathogenesis of the disease in terrestrial and aquatic mammalian species appears to be different from what is observed in birds as infection in canines (red fox) and felines (cat and bobcat [*Lynx rufus*]) often results in neurologic invasion with severe encephalitis and death (Elsmo et al., 2023). Excessive salivation, foaming at the mouth, blindness, seizures, ataxia, unconsciousness, and tremors, as well as altered behaviors such as rolling on the ground are some of the clinical signs that have been observed in foxes, cats, mink, and skunks infected with HPAI H5N1 (Alkie et al., 2023). It is important to note, however, that severely affected or dead mammals are usually the only ones to be tested, and milder cases with different clinical presentations may not be captured within current wildlife surveillance programs.

In dairy cattle, the disease and associated clinical presentation are related to the virus's tropism for the mammary gland (Burrough et al., 2024; Caserta et al., 2024; Nelli et al., 2024). Natural infections of dairy cattle with HPAI H5N1 have been associated with mild respiratory signs, lethargy, dehydration, dry/tacky feces or diarrhea, and most notably decreased milk production with milk with yellowish and thickened colostrum-like appearance (Burrough et al., 2024; Caserta et al., 2024). Initial assessments of dynamics of virus replication in affected dairy cattle revealed high levels of virus shedding in milk, with low inconsistent levels of virus shedding also detected in respiratory secretions and urine (Caserta et al., 2024). These findings were recently confirmed following experimental intramammary inoculation of dairy cattle with H5N1 B3.13 virus (Baker et al., 2024 [unpublished data]; Halwe et al., 2024 [unpublished data]). Infectious virus shedding was only detected early in infection (up to d 7–8 postinfection; Caserta et al., 2024; Halwe et al., 2024 [unpublished data]), whereas viral RNA has been detected for up 3 to 5 wk following infection (Baker et al., 2024 [unpublished data]; Caserta et al., 2024; Halwe et al., 2024 [unpublished data]). This could be a result of increasing levels of neutralizing antibodies detected in milk starting around d 7 to 8 postinfection (Baker et al., 2024 [unpublished data]; Halwe et al., 2024 [unpublished data]). Consistent with virus shedding in milk, HPAI H5N1 presents a high tropism for the mammary gland in dairy cattle (Caserta et al., 2024; Nelli et al., 2024), which correlates with the expression of SA receptors (Kristensen et al., 2024 [unpublished data]; Nelli et al., 2024) more specifically in milk-secreting epithelial cells (Nelli et al., 2024), the main target cell of the virus (Caserta et al., 2024; Nelli et al., 2024). Virus replication seems to be restricted to the quarter that was initially infected, as no virus shedding was detected in quarters that were not inoculated with the virus experimentally (Baker et al., 2024 [unpublished data]). Replication of the virus in the respiratory tract including trachea and lungs has also been demonstrated in naturally (Caserta et al., 2024; Nelli et al., 2024) and experimentally (Baker et al., 2024 [unpublished data]) infected cattle; however, extramammary replication seems to be limited (Caserta et al., 2024; Baker et al., 2024 [unpublished data]; Halwe et al., 2024 [unpublished data]). Limited virus replication has also been observed in other extra-mammary and respiratory organs, including supramammary lymph nodes, spleen, and colon (Caserta et al., 2024) (Figure 3).

**Figure 3:**
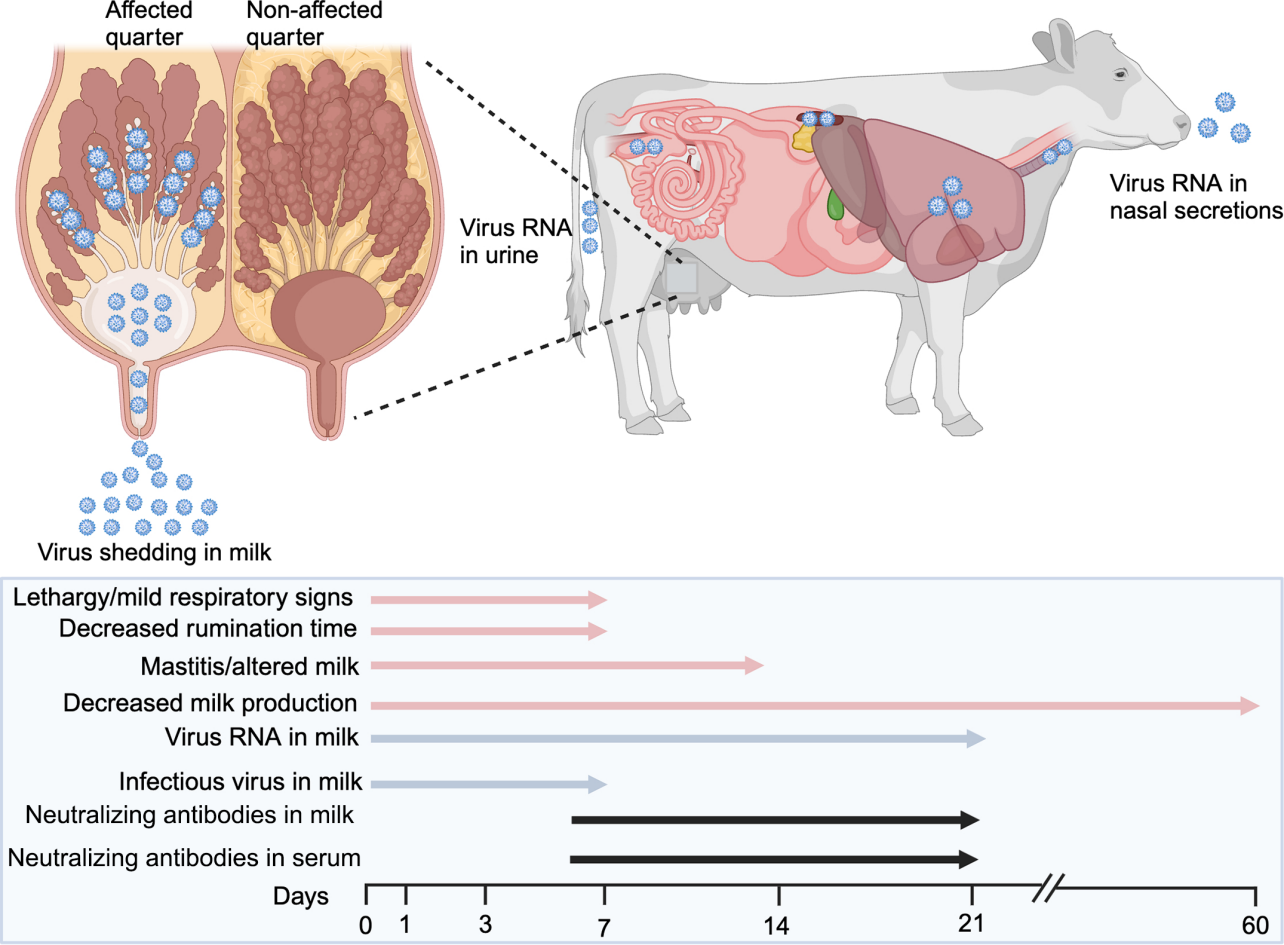
Infection dynamics, shedding, and pathogenesis of HPAI H5N1 in dairy cattle.

The main pathophysiological outcome of H5N1 virus infection in dairy cows appears to be linked to the virus replication in milk-secreting cells in the mammary gland, which leads to severe viral mastitis (Baker et al., 2024 [unpublished data]; Caserta et al., 2024; Halwe et al., 2024 [unpublished data]). Replication of the virus in these cells results in epithelial degeneration and necrosis, which in turn can lead to reduced milk production and secretion (Baker et al., 2024 [unpublished data]; Caserta et al., 2024). Reported losses vary between 10% and 100% of daily milk production, which seems to persist much longer than the clinical disease. Whether the milk losses persist in subsequent lactations of affected cows still needs to be determined.

The widespread circulation of the HPAI H5N1 clade 2.3.4.4b virus in both avian and mammalian hosts, along with its efficient transmission between cows, presents major challenges for effective disease control. The ability of HPAI H5N1 to circulate in mammalian hosts with efficient animal-to-animal transmission highlights the substantial public health threat posed by the virus. Although the number of documented instances of zoonotic transmission of the virus remains low, continued circulation of the virus in a mammalian host increases the risk of human infection. Thus, increased testing and genomic surveillance are critical to monitor virus adaptations that may lead into increased virus transmission in humans.
